# Specific T-cell immunity against Ki-ras peptides in patients with pancreatic and colorectal cancers

**DOI:** 10.1038/sj.bjc.6600697

**Published:** 2003-02-18

**Authors:** Y Shono, H Tanimura, M Iwahashi, T Tsunoda, M Tani, H Tanaka, K Matsuda, H Yamaue

**Affiliations:** 1 Second Department of Surgery, Wakayama Medical University, School of Medicine, 811-1 Kimiidera, Wakayama 641-8510, Japan

**Keywords:** mutant p21, T-cells, peptide vaccination, pancreatic cancer, colon cancer

## Abstract

Mutations of codon 12 in the *Ki-ras* gene are frequently found in pancreatic and colorectal cancers. It has been demonstrated that human T-cells have the potential to recognise tumours expressing mutated ras-derived peptides. However, it remains unclear whether T-cells from a given individual can recognise the mutant peptides, which are expressed in that individual's tumour tissues. Mutations of the *Ki-ras* oncogene were analysed by the mutant-allele-specific amplification (MASA) method in pancreatic and colorectal tumour tissues, and T-cell responses against mutated Ki-ras-derived peptides were measured by [^3^H]thymidine incorporation and IFN-γ production assays. Specific T-cell responses against Ki-ras-products were found in cancer patients, whereas no immune response was observed in normal individuals (*P*<0.01). Six of the eight pancreatic cancer patients (75%) and nine of 26 colorectal cancer patients (35%) had T-cell responses to mutated Ki-ras-derived-peptides. T-cell response in a given individual cannot recognise the same mutated ras peptide, which is expressed in that individual's tumour tissues. However, pancreatic and colorectal cancer patients have T-cell immunity against Ki-ras-peptides, and this provides potential target for cancer immunotherapy.

Malignant transformation is commonly associated with the acquisition of defined and characteristic mutations of cancer-related genes (Marx, 1989). In many circumstances, the mutations result in the expression of aberrant proteins that disrupt the normal regulation of cell growth and differentiation (Fearon and Vogelstein, 1990). Several tumour antigens have been identified and the aberrant proteins capable of inducing tumour-specific immune responses have been studied in terms of their specificity and their putative functional roles in oncogenesis (van der Bruggen *et al*, 1991; Kawakami and Rosenberg, 1997; Nestle *et al*, 1998; Rosenberg *et al*, 1998).

Somatic mutations of the *ras* genes have been shown to be involved in the malignant processes in tumorigenesis. *Ki-ras* mutations are found in approximately 90% of pancreatic adenocarcinomas and 40% of colorectal adenocarcinomas (Bos *et al*, 1987; Almoguera *et al*, 1988; Bos, 1989) These mutations convert the ras protein to a constitutively active protein, resulting in stimulation of cell proliferation. In pancreatic and colorectal cancer patients, mutated Ki-ras products are attractive targets for cancer immunotherapy since they are not commonly expressed in normal tissue and since T-cells' immune system can detect single amino-acid substitutions (Fossum *et al*, 1993).

In the present study, we have tested whether T-cells specific for mutant ras peptides from a given individual with pancreatic or colorectal cancer can recognise the same peptide expressed in that individual's tumour tissues.

## PATIENTS AND METHODS

### Patients

In total, 14 patients with pancreatic cancer, 26 patients with colorectal cancer, and six healthy volunteers from Wakayama Medical University Hospital were enrolled in this study. Informed consent for the studies was obtained from all the patients in accordance with the guideline of the Ethics Committee on Human Research, Wakayama Medical University.

### Analysis of Ki-ras mutations in codon 12

Genomic DNA was isolated from resected tumours of pancreatic and colorectal cancer patients in operation using a DNA extraction kit (QIAGEN). Approximately 3 mm^3^ of tumour materials were homogenised, and DNA extraction was performed according to the manufacturer's recommendations.

To confirm the sensitivity of the mutant-allele-specific-amplification (MASA) method, DNA extracted from two pancreatic cancer cell lines (PANC-1, BXPC3) and two lung cancer cell lines (A549, LU65) was examined for mutations of *Ki-ras* codon 12. It has been reported that PANC-1 has a *Ki-ras* 12 mutation from GGT (Gly) to GAT (Asp), BXPC3 has a wild-type allele of *Ki-ras* 12, A549 has a GGT (Gly) to (Ala) and LU65 has a GGT (Gly) to (Leu) mutation (Valenzuela and Groffen, 1986; Suwa *et al*, 1994).

### MASA method

We analysed *Ki-ras* mutations using the MASA method. This method can recognise point mutations corresponding to the 3′-end nucleotides of forward primers that have mutant 3′-end sequences (Takeda *et al*, 1993).

The *Ki-ras* mutations in codon 12 were confirmed by direct sequencing of the above PCR products.

### Peptides

The amino-acid sequences of the wild-type and mutant Ki-ras 18-mer peptides used are shown in Table 1

**Table 1:** Wild-type and mutated Ki-ras peptides (18-mers) employed in this study

**Peptide name**	**Amino-acid sequence**	**Point mutation**
*Wild-type peptide*		
Ras G 12 (p4-21)	YKLVVVGAGGVGKSALTI	(GGT)
		
*Mutated peptides*		
Ras V 12 (p4-21)	YKLVVVGAVGVGKSALTI	(GTT)
Ras C 12 (p4-21)	YKLVVVGACGVGKSALTI	(TGT)
Ras D 12 (p4-21)	YKLVVVGADGVGKSALTI	(GAT)

. The synthesis of ras-derived peptides was based on the sequence information provided by Dr MA Cheever (Qin *et al*, 1995), and purchased from TAKARA Corp., Japan. The purity of Ki-ras peptides are more than 93%.

### Cell preparation and media

Blood samples (20–40 ml) from each cancer patient and from normal individuals were obtained with informed consent. Peripheral blood mononuclear cells (PBMC) were isolated by density centrifugation over Ficoll–Paque (Pharmacia, Uppsala, Sweden). Cells were washed twice and adjusted to 2×10^6^ cells ml^−1^ in RPMI-1640 (GIBCO) containing 7% heat-inactivated autoserum, 10 mM L-glutamine, 200 U of penicillin–streptomycin ml^−1^, and 25 mM 2-mercaptoethanol.

### Lymphocyte proliferation assay

Cell aliquots suspensions (100 *μ*l) at 2×10^5^ cells ml^−1^ were plated into each well of round-bottomed 96-well microtiter plates (Corning, Corning, NY, USA). PBMC were incubated without peptides, or with 100 *μ*l of ras peptide (100 *μ*g ml^−1^), or with 100 *μ*l of phytohaemagglutinin (PHA, 5 *μ*g ml^−1^). The plates were incubated in a humidified atmosphere under 5% CO_2_/95% air at 37°C for 96 h and then incubated for 8 h with 1 *μ* Ci (35 Mbq) of [^3^H]thymidine well^−1^. Cells were then harvested, and thymidine incorporation was determined by liquid scintillation counting.

Each determination of proliferation was carried out in at least five replicated wells. The stimulation index (SI) was calculated by dividing the c.p.m. (mean) obtained from each group by the c.p.m. (mean) from autologus PBMC incubated without peptides. A positive proliferative response was defined as an SI greater than 2 (Qin *et al*, 1995).

### Interferon-*γ* (IFN-*γ*) production assay

Aliquots (2 ml) of cell suspensions at 2×10^6^ cells ml^−1^ were plated into each well of flat-bottomed, 48-well microtiter plates (Falcon No.3078). PBMC were incubated without peptides (no stimulation), or were stimulated with Ki-ras peptide (50 *μ*g ml^−1^) or PHA (5 *μ*g ml^−1^).

The plates were incubated in a humidified atmosphere of 5% CO_2_/95% air at 37°C. Primary cultures were replenished with 1 ml of fresh human interleukin-2 (IL-2)-containing inactivated autoserum medium (20 IU ml^−1^) on days 5, 10 and 15, and were stimulated with the same Ki-ras peptide (50 *μ*g ml^−1^) and with autologus PBMC (1×10^6^ cells ml^−1^), which had been inactivated with 50 *μ*g ml^−1^ of mitomycin C at 37°C for 30 min, as antigen-presenting cells, on day 10. Cultures were then continued for 20 days. Aliquots (1 ml) of the supernatants of these cultures were collected on days 5 (sup. 1), 10 (sup. 2), 15 (sup. 3), and 20 (sup. 4). The supernatants were analysed for secretion of IFN-γ using an enzyme-linked immunosorbent assay (ELISA) kit (Endogen, Boston, MA, USA). The results were expressed in IU ml^−1^. A positive response to a Ki-ras peptide was defined as production of at least 4 IU ml^−1^ of IFN-γ, which is four times greater than the production by control, in at least one of the four supernatants tested at least one time.

The HLA-DR and -DQ loci of healthy volunteers, pancreatic cancer patients and colorectal cancer patients were analysed, and the relationships between the HLA-DR and -DQ loci and positive responses of cancer patients to peptides were investigated.

## RESULTS

### Ki-ras mutation in cancer patients

Table 2

**Table 2:** Ki-ras point mutations at position 12 in pancreatic and colorectal cancer tissues

**Pancreatic cancer**	**Mutation**	**Colorectal cancer**	**Mutation**		**Mutation**
1	Gly→Asp	1	Gly→Asp	14	Wild type
2	Gly→Asp	2	Gly→Asp	15	Wild type
3	Gly→Asp	3	Gly→Asp	16	Wild type
4	Gly→Asp	4	Gly→Val	17	Wild type
5	Gly→Asp	5	Gly→Val	18	Wild type
6	Gly→Asp	6	Gly→Val	19	Wild type
7	Gly→Val	7	Wild type	20	Wild type
8	Gly→Val	8	Wild type	21	Wild type
9	Gly→Cys	9	Wild type	22	Wild type
10	Wild type	10	Wild type	23	Wild type
11	Wild type	11	Wild type	24	Wild type
12	Wild type	12	Wild type	25	Wild type
13	Wild type	13	Wild type	26	Wild type
14	Wild type				Wild type
	9/14 (64%)				6/26 (23%)

Gly=glycine; Val=valine; Asp=aspartic acid; Cys=cysteine.

shows the results of analysis of *Ki-ras* codon 12 mutations in pancreatic and colorectal cancer tissues by the MASA method. *Ki-ras* mutations were found in nine of 14 (64%) pancreatic cancer tissues and six of 26 (23%) colorectal cancer tissues. The sort of Ki-ras codon 12 mutation in tumours was aspartic acid (67%), valine (22%) and cysteine (11%) with pancreatic cancer patients, and aspartic acid (50%) and valine (50%) with colorectal cancer patients.

### Lymphocyte proliferative assay

The primary lymphocyte response to Ki-ras peptides was determined in six patients with pancreatic cancer and 11 patients with colorectal cancer. There was no enhancement of proliferation in response to Ki-ras peptides in the primary responses of any of the patients including Ki-ras mutations in their own tumours. Data were not shown.

### IFN-*γ* production assay

IFN-γ production of T-cells as a consequence of Ki-ras-derived peptide stimulation was investigated in six healthy volunteers, eight patients with pancreatic cancer and 26 patients with colorectal cancer. None of the healthy volunteers had a positive immune response against Ki-ras peptides.

### T-cell responses to Ki-ras peptides can be detected in pancreatic cancer patients

**Figure 1:**
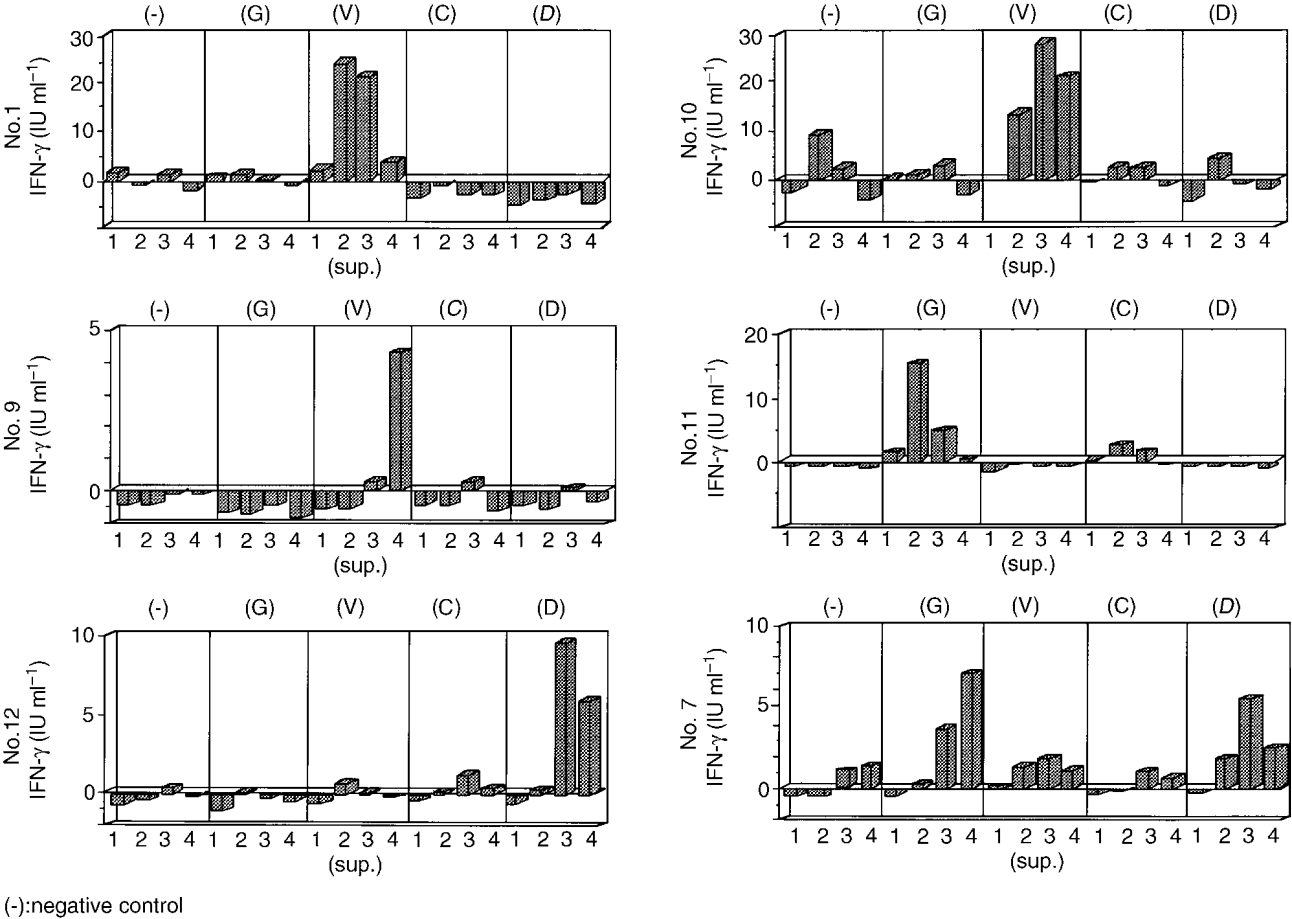
IFN-γ production in T-cell response against Ki-ras peptides in pancreatic cancer patients. IFN-γ production in pancreatic cancer patients: (1) Ki-ras mutation (D) (DR4, 9; DQ3,4); (10) Ki-ras mutation (−) (DR4, 9; DQ3, 4); (9) Ki-ras mutation (C) (DR9, 12 DQ3); (11) Ki-ras mutation (D); (12) Ki-ras mutation (−) (DR9, 8; DQ1, 3) (7) Ki-ras mutation (V) (DR8; 2 DQ1). The numbers (1–4) of supernatants indicate the period of exposure of T-cells exposed to Ki-ras peptides. Supernatants 1, 2, 3 and 4 were collected on days 5, 10, 15 and 20 after primary stimulation, respectively.

Six of the eight pancreatic cancer patients (75%) had positive responses to wild-type or mutated Ki-ras peptides (Figure 1

). Pancreatic cancer cases 1, 10 and 9 had positive responses to mutated Ki-ras V12 peptide. Pancreatic cancer case 11 had positive response to wild-type Ki-ras peptide. Pancreatic cancer case 12 had positive response to Ki-ras D12 peptide. Pancreatic cancer case 7 had a positive response to wild-type and Ki-ras D12 peptides. *Ki-ras* mutations were analysed in pancreatic cancer tissues from the patients, and results showed that cases 1 and 11 had D12 mutations, case 9 had a C12 mutation, case 7 had a V12 mutation, and cases 10 and 12 had no mutation.

### T-cell responses to Ki-ras peptides can be detected in colorectal cancer patients

**Figure 2:**
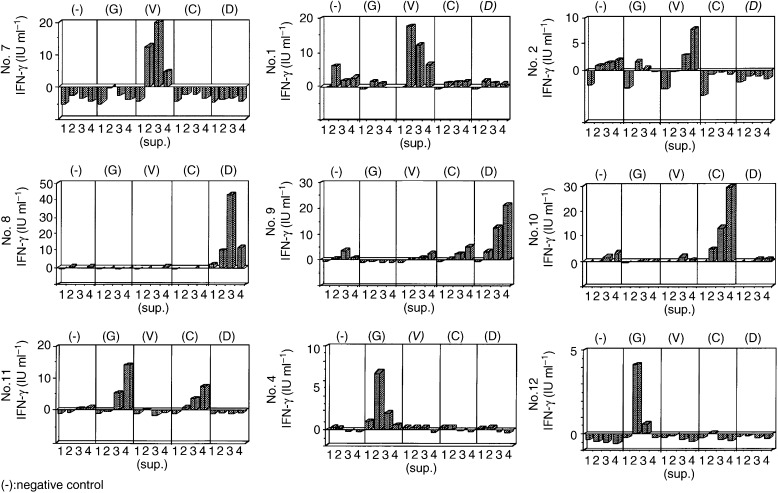
IFN-γ production in T-cell response against *Ki-ras* peptides in colorectal cancer patients. IFN-γ production in colorectal cancer patients: (7) Ki-ras mutation (−) (DR4, 6; DQ1, 4) (1) Ki-ras mutation (D) (DR1, 8; DQ1) (2) Ki-ras mutation (D); (8) Ki-ras mutation (−) (DR8, 12; DQ3, 4) (9) Ki-ras mutation (−) (DR2,6; DQ1); (10) Ki-ras mutation (−) (DR2,6; DQ1,3); (11) Ki-ras mutation (−) (DR1, 4; DQ1, 3)∼ (4) Ki-ras mutation (V) (DR8; DQ1, 3); (12) Ki-ras mutation (−) (DR6; 9 DQ1). The numbers (1–4) of supernatants indicate the period of exposure of T-cells exposed to Ki-ras peptides. Supernatants 1, 2, 3 and 4 were collected on days 5, 10, 15 and 20 after primary stimulation, respectively.

Nine of 26 colorectal cancer patients (35%) had positive responses to wild-type or mutated Ki-ras peptides (Figure 2

). Colorectal cancer cases 7, 1 and 2 had positive responses to mutated Ki-ras D12 peptide. Colorectal cancer cases 8 and 9 had positive responses to wild-type Ki-ras C12 peptide. Colorectal cancer cases 4 and 12 had positive response to wild-type peptide. Colorectal cancer case 11 had positive response to mutated Ki-ras C12 and wild-type peptides. *Ki-ras* mutations were analysed in colorectal cancer tissues from the patients, and results showed that cases 1 and 2 had D12 mutations, case 4 had a C12 mutation, and cases 7–12 had no mutation.

Thus, specific T-cell immunity against Ki-ras products was present in cancer patients, whereas no immune response was observed in healthy volunteers (*P*<0.01). Six of the eight pancreatic cancer patients and nine of the 26 colon cancer patients had a response to Ki-ras peptides. However, the T-cells immunity of a given individual could not recognise the mutant ras peptide that was expressed in the tumour tissues of that individual (Table 3

**Table 3:** T-cell response against K-ras peptides

**Healthy volunteers**					**Colorectal cancer patients**				
**No.**	**DR, DQ Locus**	**Peptides (which sort of response)**			**No.**	**DR, DQ locus**	**Tumor tissue Ki-ras mut.**	**Peptides (which sort of response)**	
1	DR4 DQ4, 7	None	(T-cell response 0/10 (0%))		7	DR4, 6 DQ1, 4	Wild type	Val	
2	DR8, 10 DQ1, 9	None			1	DR1, 8 DQ1	Gly→Asp	Val	
3	DR4, 8 DQ1, 4	None			2	NT	Gly→Asp	Val	
4	DR9, 8 DQ3, 1	None			8	DR8, 12 DQ3, 4	Wild type	Asp	
5	DR4, 9 DQ3, 4	None			9	DR2, 6 DQ1	Wild type	Asp	
6	DR8, 9 DQ1, 3	None			10	DR2, 6 DQ1, 3	Wild type	Cys	
7	DR10, 2 DQ1	None			11	DR1, 4 DQ1, 3	Wild type	Gly, Cys	
8	DR4, 9 DQ3, 4	None			4	DR8 DQ1, 3	Gly→Val	Gly	
9	DR2, 8 DQ1, 6	None			12	DR6, 9 DQ1, 3	Wild type	Gly	
10	DR8, 4 DQ1, 4	None			3	DR6, 8 DQ1	Gly→Asp	None	
**Pancreatic cancer patients**					7	NT	Gly→Val	None	
**No**	**DR, DQ Locus**	**Tumor tissue Ki-ras mut.**	**Peptides (which sort of response)**		6	DR1, 4 DQ1, 3	Gly→val	None	
1	DR4, 9 DQ3, 4	Gly→Asp	Val		13	NT	Wild type	None	
10	DR4, 9 DQ3, 4	Wild type	Val	(T-cell	14	DR4, 8 DQ1, 4	Wild type	None	
9	DR9, 12 DQ3	Gly→Cys	Val	response 6/8	15	DR2, 4 DQ1, 2	Wild type	None	
11	NT^a^	Wild type	Gly	(75%)2)	16	DR1, 6 DQ1, 3	Wild type	None	
12	DR9, 8 DQ1, 3	Wild type	Asp		17	DR8, 9 DQ1, 3	Wild type	None	
7	DR8, 2 DQ1	Gly→Val	Asp. Gly		18	DR4, 8 DQ1, 4	Wild type	None	
2	DR10, 9 DQ1, 3	Gly→Asp	None		20	DR4, 9 DQ3, 4	Wild type	None	
13	DR4, 9 DQ3, 4	Wild type	None		21	DR4, 6 DQ1, 4	Wild type	None	(T-cell response 9/26 (35%)*)
					22	DR4, 6 DQ1, 4	Wild type	None	
					23	DR1, 4 DQ1, 3	Wild type	None	
					24	DR2, 4 DQ1, 4	Wild type	None	
					25	DR6, 8 DQ1, 3	Wild type	None	
					26	NT	Wild type	None	

a NT=not tested; Gly=glycine; Val=valine; Asp=aspartic acid; Cys=cysteine.

* *P*<0.001, compared to normal individuals.

).

### Relationship between HLA-DR and -DQ loci and positive response against Ki-ras peptides in cancer patients

HLA-DR and -DQ of healthy volunteers, pancreatic cancer patients, and colorectal cancer patients were examined. Among five patients who had positive responses to Ki-ras V12 peptide, three patients had DR4, DR9, DQ3 or DQ4 locus. Among four patients who had positive responses to Ki-ras D12 peptide, three patients had DR8 or DQ1 locus. The two patients who had positive responses to Ki-ras C12 peptide were found to have DQ1 or DQ3 locus. Among four patients who had positive responses to wild-type peptide, two patients had DQ1 or DQ3 locus (Table 4

**Table 4:** Relation between HLA-DR and - DQ locus and positive response to peptides in cancer patient

	**HLA**			
**Patient No.**	**-DR Locus**	**-LR Locus**	**Mutation in tumour tissues**	**Responding peptides**
P1	DR4, 9	DQ3, 4	Gly→Asp	Val
C1	DR1, 8	DQ1	Gly→Asp	Val
P9	DR9, 12	DQ3	Gly→Cys	Val
P10	DR4, 9	DQ3, 4	Wild type	Val
C7	DR4, 6	DQ1, 4	Wild type	Val
P7	DR2, 8	DQ1	Gly→Asp	Asp, Gly
C8	DR8, 12	DQ3, 4	Wild type	Asp
C9	DR2, 6	DQ1	Wild type	Asp
P12	DR8, 9	DQ1, 3	Wild type	Asp
C10	DR2, 6	DQ1, 3	Wild type	Cys
C11	DR1, 4	DQ1, 3	Wild type	Cys, Gly
C4	DR8	DQ1, 3	Gly→Val	Gly
P7	DR2, 8	DQ1	Gly→Asp	Gly, Asp
C12	DR6, 9	DQ1, 3	Wild type	Gly
C11	DR1, 4	DQ1, 3	Wild type	Gly, Cys

		**DR**							**DQ**		
**Peptide**	**Total no.**	**1**	**2**	**4**	**6**	**8**	**9**	**12**	**1**	**3**	**4**
Val	5	1		3	1	1	3	1	2	3	3
Asp	4		2		1	3	1	1	3	2	1
Cys	2	1	1		1				2	2	
Gly	4	1	1	1	1	2	1		4	3	

P=pancreatic cancer patients; C=colon cancer patients.

).

## DISCUSSION

Activating amino-acid substitutions impair the intrinsic GTPase activity of the ras protein and generate constitutively activated signal complexes with transforming activity. Point mutations in *ras* genes have been found in a wide variety of human and murine cancers, especially in human pancreatic cancers (90%) and human colorectal cancers (45%) (Bos *et al*, 1987; Almoguera *et al*, 1988; Bos, 1989).

A large fraction of human cancers harbour point mutations in the *ras* gene at codon 12, in which the normal residue is substituted by a Val, Asp or Cys residue. From an immunological perspective, these determinants may represent highly specific epitopes for T-cell (CD4+ and/or CD8+) recognition in cancer immunotherapy (Tsang *et al*, 1994; Fossum *et al*, 1995). Evaluation of point-mutated *ras* as a T-cell epitope could be determined biologically using short synthetic peptides that precisely correspond to the altered sites.

Several laboratories established approaches in both murine and human systems to evaluate point-mutated ras p21 oncogene products as potential tumour-specific targets and to characterise the resulting cellular immune responses.

Studies using those system have shown that mutant ras protein is able to serve as a tumour-specific antigen (Peace *et al*, 1991a; Cheever *et al*, 1995). T-cells from animals immunised by ras peptides can lyse cells transformed to express the ras products with the same mutation in animal models (Peace *et al*, 1991b).

The observation that ras products can be immunogenic in mice suggested that similar T-cell responses might be present in humans (Peace *et al*, 1991a). *In vitro* stimulation of human T-cells from some normal individuals or cancer patients with mutant ras peptides results in the expansion of CD4+ and CD8+ precursors, which may exhibit cytotoxicity against autologous or MHC-matched, antigen-bearing target cells (Fossum *et al*, 1994b; Juretic *et al*, 1996). In addition, humoral responses specific for ras products have been observed in colorectal cancer patients (Takahashi *et al*, 1995).

Other studies showed that the human T-cells can recognise peptides that span the mutated segment of the ras protein and that the *ras* peptide-specific T-cells can respond to ras protein containing the same substitution (Jung and Schluesener, 1991; Gedde-Dahl *et al*, 1992; Fossum *et al*, 1994a; Qin *et al*, 1995). Previous studies have identified that *ras* mutation-specific memory T-cells in only two human cancer patients with follicular thyroid cancer and colorectal cancer (Gedde-Dahl *et al*, 1992; Fossum *et al*, 1994a,1994b). In both of these cases, the corresponding *ras* mutation could not be detected in the available tumour biopsy samples.

However, it remains unclear whether T-cells can recognise the same mutated peptides that are expressed in tumour tissues from the same individual tissue. We first analysed the correlation between the peptides that induce T-cell response against Ki-ras peptides and the *Ki-ras* mutations in pancreatic and colorectal cancer tissues.

We are not able to show that T-cells can recognise the mutated ras peptide that is expressed in the tumour cells from the same individuals.

To assess the immune response against Ki-ras peptides in patients with pancreatic and colorectal cancer patients, primary proliferative response assays and IFN-γproduction assays were done. No effect of these peptides could be detected using the primary proliferative response assay, because of the low immunogenicity of the Ki-ras products. On the other hand, it was possible to detect a Ki-ras response by twice stimulation with Ki-ras peptides and stimulation by IL-2 in an IFN-γ production assay. The stimulation of such a low concentration of IL-2 is intended to suppress the secretion of IFN-γ from other cell sources, including the activation of natural killer cells. The purpose of IFN-γ production assays is to determine which peptide is significant for the T-cell activation, and actual concentration is not calculated. We used the 18-mer peptides that can bind to class II molecule, but not to class I molecule. Therefore, the CD4+ cells might be related in this study. However, the primary culture in the IFN-γ production assay contains antigen-presenting cells, so CD8+ cells may be contributed in this assay. T-cell immunity against Ki-ras peptides was detected at lower frequency in colorectal cancer patients than in pancreatic cancer patients. This suggested that the immunogenicity to ras products was recognised more strongly in pancreatic cancer patients who had a high frequency of *ras* mutations in their tumour tissue, but the T-cells did not recognise the ‘correct’ mutation. T-cells in the patients with pancreatic cancer may have been previously exposed to similar antigens *in vivo*.

We propose two possible reasons why T-cells from a given individual cannot recognise the same mutated ras peptide expressed in the tumour tissues of that individual.

First, the length of the peptides bound by MHC class II molecules are not strongly constrained. Therefore, the binding of peptides to MHC class II molecules is more promiscuous than the binding of peptides to MHC class I molecules. Peptides that bind to MHC class II molecules are variable in length and their anchor residues lie at various distances from the end of the peptide (Rudensky *et al*, 1991; Rammensee *et al*, 1995; Hammer *et al*, 1994). In patients who respond to wild-type peptide, their anchor motif of class II molecule are considered to be similar to wild-type peptide.

Second, we speculate that tumour cells harbouring a mutation have been eliminated by the immune system in cancer patients. *Ki-ras* mutations are thought to occur as a relatively early event in the developmental sequence of colorectal adenocarcinoma (Vogelstein *et al*, 1988), and may therefore be expressed early on by most of the tumour cells. The cancer present at the time of biopsy may have progressed further, and may not harbour the same mutation because the cancer cells harbouring the earlier mutation were eliminated by the immune system in an early event of tumour development (Nakagawa *et al*, 1991).

It is suggested that T-cells responding to synthetic 18-mer Ki-ras peptides are restricted by HLA-DR or -DQ class II molecules. Ras p21 is an internally localised biosynthetic protein and is therefore potentially susceptible to the endogenous pathway of antigen processing and subsequent loading with MHC class I or II molecules. It is possible that point-mutated ras p21 proteins may be processed through an exogenous mechanism. In this regard, Harmer *et al* reported that point-mutated ras p21 proteins can be found in the external tumour microenvironment as well as in the plasma of tumour-bearing mice, and if so, may be available as exogenous antigens for endosomal processing by antigen-presenting cells and presentation to CD4+ T-cells (^Harmer *et al*, 1991^).

CD4+ T-cells are thought to play an important and central role in immunoregulation through the production and action of lymphokines. Accordingly, several peptide- or protein-based immunotherapy has great therapeutic potential for cancer patients whose tumours harbour point mutations in codon 12 of the ras p21 proto-oncogene.
